# Improved Binding Affinity of Omicron’s Spike Protein for the Human Angiotensin-Converting Enzyme 2 Receptor Is the Key behind Its Increased Virulence

**DOI:** 10.3390/ijms23063409

**Published:** 2022-03-21

**Authors:** Rajender Kumar, Natarajan Arul Murugan, Vaibhav Srivastava

**Affiliations:** 1 Division of Glycoscience, Department of Chemistry, School of Engineering Sciences in Chemistry, Biotechnology and Health, KTH Royal Institute of Technology, AlbaNova University Center, 106 91 Stockholm, Sweden; rajkum@kth.se; 2 Department of Computer Science, School of Electrical Engineering and Computer Science, KTH Royal Institute of Technology, 100 44 Stockholm, Sweden

**Keywords:** severe acute respiratory syndrome coronavirus type 2 (SARS-CoV-2), Omicron, human angiotensin-converting enzyme 2 (hACE2), molecular dynamics simulation, receptor-binding domain (RBD), receptor-binding motif (RBM), molecular mechanics-generalized Born surface area (MM-GBSA)

## Abstract

The new variant of severe acute respiratory syndrome coronavirus type 2 (SARS-CoV-2), Omicron, has been quickly spreading in many countries worldwide. Compared to the original virus, Omicron is characterized by several mutations in its genomic region, including the spike protein’s receptor-binding domain (RBD). We have computationally investigated the interaction between the RBD of both the wild type and Omicron variant of SARS-CoV-2 with the human angiotensin-converting enzyme 2 (hACE2) receptor using molecular dynamics and molecular mechanics-generalized Born surface area (MM-GBSA)-based binding free energy calculations. The mode of the interaction between Omicron’s RBD with the hACE2 receptor is similar to the original SARS-CoV-2 RBD except for a few key differences. The binding free energy difference shows that the spike protein of Omicron has an increased affinity for the hACE2 receptor. The mutated residues in the RBD showed strong interactions with a few amino acid residues of hACE2. More specifically, strong electrostatic interactions (salt bridges) and hydrogen bonding were observed between R493 and R498 residues of the Omicron RBD with D30/E35 and D38 residues of the hACE2, respectively. Other mutated amino acids in the Omicron RBD, e.g., S496 and H505, also exhibited hydrogen bonding with the hACE2 receptor. A pi-stacking interaction was also observed between tyrosine residues (RBD-Tyr501: hACE2-Tyr41) in the complex, which contributes majorly to the binding free energies and suggests that this is one of the key interactions stabilizing the formation of the complex. The resulting structural insights into the RBD:hACE2 complex, the binding mode information within it, and residue-wise contributions to the free energy provide insight into the increased transmissibility of Omicron and pave the way to design and optimize novel antiviral agents.

## 1. Introduction

After its emergence in 2019, SARS-CoV-2 (severe acute respiratory syndrome coronavirus type 2) has rapidly affected the worldwide population and caused severe pandemics [1,2]. The four main structural proteins present in coronaviruses are the spike (S), envelope (E), nucleocapsid (N), and membrane (M) proteins [2,3]. The primary function of the S protein is to bind to the receptor angiotensin-converting enzyme 2 (ACE2) that helps the virus enter the host cell [4]. Thus, the S protein plays a critical role in the initial stage of infection for disease-causing coronaviruses. Therefore, studies focused on understanding the detailed mechanism of S protein-ACE2 interactions are crucial. This protein-protein complex is considered a prime target for developing new drugs and vaccines. Several SARS-CoV-2 variants have been reported thus far around the world. Based on the evidence of their increased transmissibility, disease severity, and/or possible reduced vaccine efficacy, four variants have been universally categorized as variants of concern (VOCs) [5]. Zhang et al. (2021) showed that, compared to the original virus, the D614G mutation makes the spike protein more stable in the new SARS-CoV-2 variants, resulting in higher contagiousness [6]. The same research group later reported the structure, function, and antigenicity of the full-length spike protein trimer of the Delta variant and compared the results with the other five SARS-CoV-2 variants [7]. They concluded that the increased transmissibility of the Delta variant, compared to the other variants, could be because of its efficient binding to the cellular receptor ACE2 and faster infection rate in the target cells [7]. 

A new SARS-CoV-2 variant (belonging to the Pango lineage B.1.1.529) was identified in South Africa at the end of November 2021 [8]. The World Health Organization (WHO) named this variant Omicron and classified it as a VOC. Based on the changes that occurred at the sequence level in the Omicron variant, it is assumed that it may be more transmissible than the original SARS-CoV-2 virus and other variants (e.g., Alpha, Delta, etc.); however, no in-depth study based on experimental and computational methods has supported this assumption. Nevertheless, the number of SARS-CoV-2 cases has increased worldwide and poses a major threat to healthcare systems worldwide. Recently, researchers from the University of Hong Kong explained the reason behind the increased transmissibility of Omicron as compared to previous SARS-CoV-2 variants [9]. They showed that Omicron infects and multiplies 70 times faster than the original virus and its Delta variant in the human bronchus. Grabowski et al. (2021) reported the exponential growth of Omicron over four weeks (8 November–5 December 2021), with an estimated doubling time of 3.38 days [10]. Indeed, WHO has also recognized that Omicron is spreading at an exceptional rate [11].

**Figure 1:**
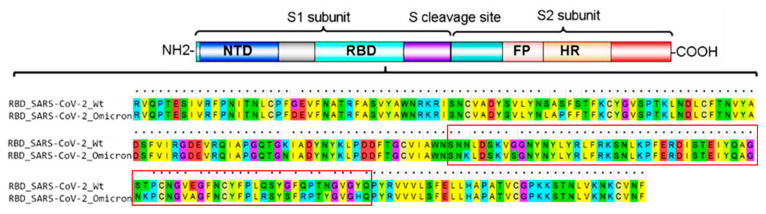
The coronavirus spike protein domain architecture and sequence alignment of the original RBD of SARS-CoV-2 and the RBD of the Omicron variant. Asterisks denote identical amino acid residues. The red rectangular box indicates the receptor-binding motif of RBD. NTD, N-terminal domain; RBD, receptor-binding domain; FP, fusion peptide; HR, heptad repeat.

It was found that compared to the original wild type (wt) virus, the Omicron variant is characterized by 30 amino acid mutations, three small deletions, and one small insertion in the spike protein [8]. Apparently, 15 of these mutations are located in the receptor-binding domain (RBD). Of these, 10 mutations are specific to the receptor-binding motif (RBM) (Figure 1), through which the viral spike protein interacts directly with ACE2 receptors.

In addition, the Omicron variant carries some changes and deletions in other genomic regions [8]. The initial step of the infection process is the binding of the spike protein RBD to the human ACE2 (hACE2) receptor, indicating that viral transmissibility and virulence largely depend on the interaction between these proteins. Mutations in the RBD of the spike protein or other surface structures can change the new variant’s antigenic properties, reducing the neutralization activity of antibodies and resulting in a higher risk of reinfection or decreasing vaccines effectiveness. Therefore, there is an urgent need to study the mutation pattern of the Omicron SARS-CoV-2 variant and its mechanism of virulence or pathogenesis to develop effective countermeasures. In this study, we have investigated how different mutations in the RBD of the spike protein of the Omicron variant affect its binding to the ACE2 receptor. A fundamental understanding of the sequence to structural level interactions between the Omicron spike RBD and hACE2 is necessary in developing new treatments for coronavirus infections.

## 2. Result and Discussion

**Figure 2:**
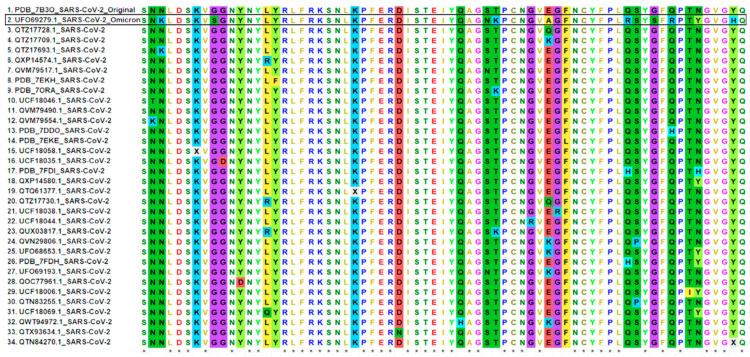
Comparative sequences analysis of the spike RBM of SARS-CoV-2. A total of 34 representative sequences were aligned. Asterisks denote identical amino acid residues.

The structural information of the SARS-CoV-2 RBD:hACE2 complex (PDB ID: 7A91) has been reported in the PDB database (https://www.rcsb.org/, accessed on 10 December 2021) that provides in-depth information about their binding interface. A receptor-binding motif (RBM, about 69 amino acids) of the RBD directly contacts and binds to the ACE2 receptor, clearly indicating that any mutation in the RBM might play a vital role in the infection process [12]. Indeed, many naturally occurring mutations in SARS-CoV-2, mainly those in the RBD, affect its binding to the ACE2 receptor [13]. Here, we performed a comparative sequence analysis to examine how the key residues changed over time within the RBM of all reported SARS-CoV-2 strains (Figure 2). It was found that, in total, 22 different positions (Figure 2) were substituted with various amino acids in the RBM of SARS-CoV-2 variants, including the Omicron variant. In particular, the Omicron RBM has a total of 10 mutations: N440K, G446S, S477N, T478K, E484A, Q493R, G496S, Q498R, N501Y, and Y505H (Figure 2). Apparently, the residues E484 and N501 (as in wt-SARS-COV-2) were substituted many times by different amino acids in different variants (Figure 2). In the case of Omicron, these amino acids were mutated with alanine and tyrosine (E484A and N501Y). In addition, six mutations at positions G446S, E484A, Q493R, G496S, Q498R, and Y505H were observed only in Omicron, while the rest of the four mutations were also present in other variants (Figure 2). The six unique mutations mentioned above in the Omicron RBM may directly affect the binding of the spike protein to the host cell ACE2 receptor.

Niu et al. (2021) reported that the RBM Q498H substitution found in pangolin CoVs enhanced the binding capacity of such RBMs to the ACE2 receptor homologs of mice, rats, and European hedgehogs [14]. The glutamine residue at this position is replaced by arginine in the Omicron variant (Figure 2); however, the effect of this crucial mutation has not been studied thus far computationally. The Q498R mutation has been shown to make hydrogen bonds and salt bridges with ACE2 (the residues involved are Q42 and D36) in a recent study based on cryo-electron microscopy [15]. A similar type of study, as performed by Niu et al. (2021), can also be informative for investigating the effect of this mutation on ACE2 homologs in other hosts.

To better understand the structural aspects of the Omicron RBD binding mode to the ACE2 receptor, we have performed computational modelling and large-scale molecular dynamics simulation studies for Omicron-RBD:hACE2 and wt-RBD:hACE2 complexes. We observed that the overall protein complex was stable except for some flexibility in the loop regions during the MD simulation. The computed root mean square fluctuation (RMSF), which is proportional to the thermal factor (Figure S1a), shows that the conformational flexibility was not altered significantly in the case of the Omicron variant when compared to the wild type. This was also the case for the hACE2 receptor in these two complexes (Figure S1b). The binding free energies computed for the spike RBD with the hACE2 receptor for the wild type and the Omicron variant are listed in Table 1. It was observed that the binding free energy for the Omicron spike with the hACE2 receptor was lower than that of the wt spike by −8.6 kcal/mol. This indicates an increased binding affinity towards the hACE2 receptor for the Omicron variant spike protein, which can be directly associated with its increased infection rate. Further observations (Table 1) suggest that the major driving force for the increased binding affinity can be attributed to the increased electrostatic and hydrophobic interactions. Indeed, in the case of the Omicron spike, the electrostatic interaction energy difference was almost double when compared to the wt spike. However, the increased binding potential due to electrostatic contributions is largely compensated by the polar solvation free energies, which are comparably larger for the Omicron variant. The sum of the electrostatic and polar solvation energy is, in fact, more positive for the Omicron variant, suggesting that the stabilization is largely due to hydrophobic interactions. The difference in van der Waals interactions was −15.6 kcal/mol (Table 1) between Omicron and wild type strains.

**Figure 3:**
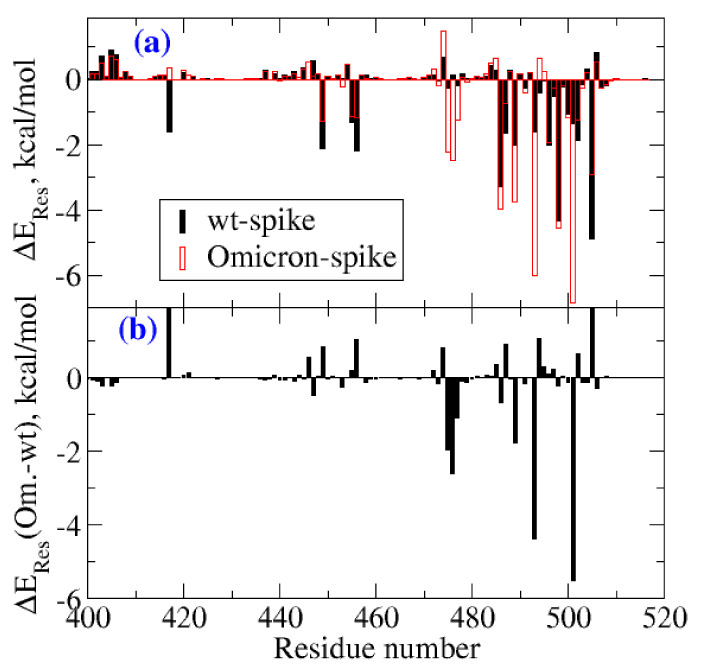
(**a**) Residue-wise contributions for the binding free energies. The results are shown only for residues in the RBD of the spike protein. Further, the numbering of residues is shifted by three for the Omicron to account for the three deletions. (**b**) The difference in residue-wise contributions to the binding free energies between the spike proteins of the Omicron variant and the wild type (wt).

In the Figure 3, the subplot 3a shows the residue-wise decomposition of the binding free energies for the RBD domain of the wild type virus and the Omicron variant, while subplot 3b shows the difference between these two results. A positive sign refers to destabilizing residues in the case of Omicron, while the negative sign shows the stabilizing residue-wise contribution. The analysis showed that residues 475–477, 489, 493, and 501 contributed to the increased binding potential of the Omicron variant. Each of these residues contributed to binding free energies lower than −1.0 kcal/mol. In particular, the residues 476, 493, and 501 contributed with −2.61, −4.38, and −5.49 kcal/mol. As we will see, the major contributions were due to hydrophobic, electrostatic, and hydrogen-bonding interactions. Further, in order to understand the increased binding affinity, the residue-level contributions, hydrogen bonding, and salt-bridges were analyzed between the protein-protein complexes.

In the Omicron-RBD:hACE2 complex, a total of 11 significant hydrogen bonds and 3 different salt bridge ion pairs were observed. The four key hydrogen bonds, T500-D355, G502-K353, R493-D30, and R498-D38, were found with occupancies of 54.20%, 48.60%, 39.20%, and 28.80%, respectively (Table 2). In the wt-RBD:hACE2 complex, a total of 14 hydrogen bonding interactions and 1 salt bridge ion pair were observed. We also observed that some hydrogen bonding interactions were similar in both complexes with different hydrogen bond occupancies. In the wt-RBD:hACE2 complex, the highest hydrogen bond occupancy, 70.50%, was observed for the Y449–D38 pair, while the value for the same pair in Omicron was only 17.10% (Table 2). This lower occupancy is because, in the Omicron RBD:hACE2 complex, D38 of hACE2 is also occupied by two residues of the Omicron RBD, the mutated R498 via a salt bridge interaction and another mutated S496 via a hydrogen bond. The hydrogen bond between G502–K353 was found with an occupancy of 48.60% in both the wild type and Omicron complexes.

**Figure 4:**
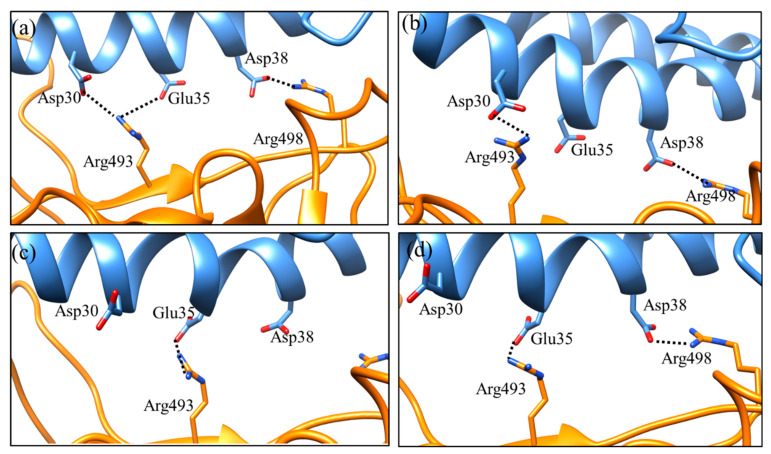
The electrostatic interaction involving residues and their interactions observed during MD simulation: (**a**) All possible salt bridge pairs Arg493 -- Asp30, Arg493 -- Glu35, and Arg498 -- Asp38 observed during the entire 100 ns MD simulation, (**b**) the salt bridge pairs Arg493 -- Asp30 and Arg498 -- Asp38 observed between 0 and 70 ns of MD simulation, (**c**) the salt bridge pair Arg493 -- Glu35 observed between 80 and 100 ns of MD simulation, and (**d**) the salt bridge pairs Arg493 -- Glu35 and Arg498 -- Asp38 observed for a very short time between 80 and 85 ns of MD simulation. The hACE2 receptor is shown in blue and the spike RBD by orange.

A key interacting interface glutamine residue present at two different positions, 493 and 498, in the wild type was mutated to a positively charged amino acid arginine in the Omicron variant (Figure 2, Table 2). These mutated residues play an important role in the binding interaction of the RBD:hACE2 complex not only via hydrogen bonds but also through salt bridges (Figure 4). It is worth mentioning that these interactions were consistently observed during the entire MD simulations. These salt bridges did not occur in the wt-RBD:hACE2 complex, mainly because of the glutamine residues at these positions. Other hydrogen bond-forming residues in the RBD:hACE2 complex were found with lower occupancies, and some of them are mutated in the Omicron variant; for example, residues pairs H505–A386, and S496–D38. Altogether, the mutated residues resulted in a stronger interaction between the Omicron RBD and hACE2 via hydrogen bonds and salt bridges.

It has been reported that in the original SARS-CoV-2, the residue K417 forms a salt bridge and hydrogen bond with D30 of hACE2 [1]. However, in Omicron, the K417 is mutated to an asparagine residue, resulting in the loss of the electrostatic interaction with Asp30 of the hACE2 receptor. The same mutation (K417N) in the Delta variant of SARS-CoV-2 was associated with a slight decrease in the ACE2-binding affinity [16]. Detailed information about the hydrogen bonding of these residues and their occupancies and electrostatic interactions in ion pairs is given in Table 2.

**Figure 5:**
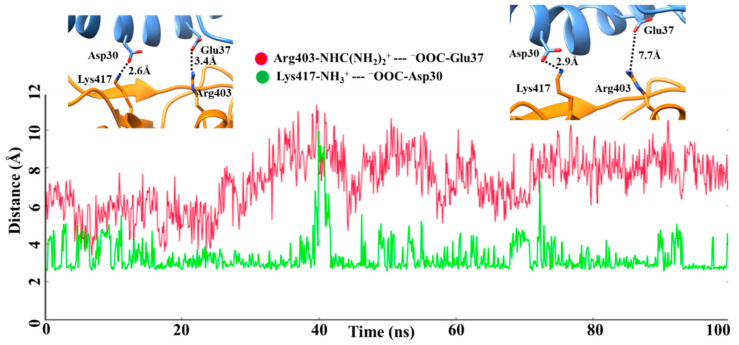
Salt bridges interactions and their corresponding distances between the wild type spike RBD and the hACE2 receptor complex during 100 ns of MD simulation.

Interestingly, some key differences were found when comparing the salt bridge interactions in both complexes. In the wt-RBD:hACE2 complex, only one salt bridge between Lys417-NH_3_^+^ -- ^−^OOC-Asp30 was consistently formed with an average distance of approximately 2.9 Å during the MD simulation (Figure 5). Only in a few conformations did the ion pair Arg403-NHC(NH_2_)_2_^+^ -- ^−^OOC-Glu37 come closer, as seen in Figure 5, and after a while, it showed a greater distance, more than 7.0 Å. For the wt-RBD:hACE2 complex, we considered only one salt bridge, K417–D30, which is also reported in the crystal structure [1]. While in the case of the Omicron-RBD:hACE2 complex K417 is mutated to glutamine, this did not interact with hACE2 residues. As mentioned earlier, the mutated residues Q493R and Q498R have large side chains with positively charged functional groups that showed electrostatic interactions with the negatively charged amino acids D30, E35, and D38 of the hACE2 receptor (Figure 4, Table 2).

**Figure 6:**
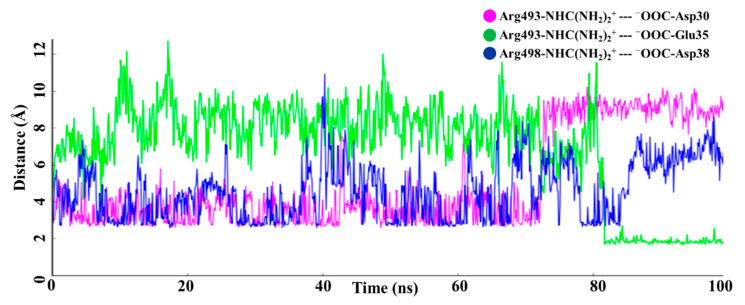
The distance between residues participating in the salt bridge (electrostatic interaction) during the entire MD simulation.

We observed two salt bridges which consistently formed during the MD simulation, via the ion pairs Arg493-NHC(NH_2_)_2_^+^ -- ^−^OOC-Asp30/-Glu35 and Arg498-NHC(NH_2_)_2_^+^ -- ^−^OOC-Asp38 (Figure 4). As shown in Figure 6, the salt bridge pairs R493 -- D30 and R498 -- D38 were observed between 0 and 70 ns of MD simulation, while the salt bridge pair R493 -- E35 (average distance less than 2 Å) was observed between 80 and 100 ns of MD simulation (Figure 6). The side chain of residue R493 showed two different conformations; one was observed near D30 for a longer time, while the alternate conformation closer to E35 was observed for short times during MD simulations. Two very short lifetime salt bridge pairs, R493 -- E35 and R498 -- D38, were simultaneously observed between 80 and 85 ns of MD simulation (Figure 6).

Additionally, a non-canonical π–π stacking interaction was found at the interface of the Omicron RBD:hACE2 complex between two tyrosine residues located on the protein surface. The aromatic ring of the mutated Y501 residue in the Omicron RBD showed a pi-stacking interaction with the aromatic ring of residue Y41 of hACE2. It was found that the geometry of this interaction was T-shaped, where the aromatic rings of Y41 and Y501 were perpendicular to each other (Figure S2). It has been previously shown that such Tyr−Tyr interactions occur mainly at the protein surface [17]. The time evolution of the center of mass distance of the two tyrosine residues and the distance between the center of mass of one tyrosine with the nearest hydrogen atom (CH) in the phenyl ring of the second tyrosine is shown in the Supplementary Figure S3. The average distance (between the center of mass of phenyl group of one tyrosine and CH group of another tyrosine) obtained in most of the conformations was below 5 Å between aromatic rings. Even though the pi-pi stacking interactions are best described by symmetry-adapted perturbation theory, the van der Waals term in the force-field captures this interaction fairly well. As shown in Figure S3, the contact is maintained in most configurations during the 100 ns simulation, suggesting that this can be one of the strongest interaction hotspots between the spike protein and the human ACE2 receptor, stabilizing the protein-protein complex. As McGaughey (1998) previously analyzed, aromatic amino acid side chains can stabilize interactions in proteins with a greater distance than the average van der Waals radii [18]. The residue-wise decomposition analysis of binding free energies showed that residue 501 of the spike protein of the Omicron variant is the one dominantly contributing, with as much as −5.5 kcal/mol. The mutation N501Y in the RBD reported for SARS-CoV-2 variants has been shown to increase ACE2 binding affinity due to improved π–π stacking [15,19] and is also associated with increased infection and transmission [20]. In the Omicron-RBD:hACE2 complex, both tyrosine residues are located on the protein’s surface. This interaction is absent in the wt-RBD:hACE2 complex as the amino acid present at position 501 in wt-RBD is an asparagine that is mutated to tyrosine in Omicron.

As demonstrated in this study, the force-field based molecular dynamics and binding free energy calculations can provide insight into changes in binding affinity/potential due to mutations in the spike protein with the hACE2 receptor. With this knowledge, the highly virulent nature of virus variants can be predicted, and the healthcare system can be alerted regarding the risk associated with their eruptions/spread. As a continuation of this work, experimental validation could be performed by binding assay studies on expressed spike proteins with the receptor.

## 3. Materials and Methods

### 3.1. Sequence Analysis and Structure Modelling

For comparative analysis, the sequences of the receptor-binding motif (RBM) from all reported SARS-CoV-2 variants were retrieved from the NCBI database using the BlastP program (accessed on 15 December 2021). Further, sequence redundancy was removed at 100% sequence identity, and the remaining representative sequences were aligned using the EBI-MUSCLE program (https://www.ebi.ac.uk/Tools/msa/muscle/, accessed on 16 December 2021). For computational modelling, all 15 RBD substitutions G339D, S371L, S373P, S375F, K417N, N440K, G446S, S477N, T478K, E484A, Q493R, G496S, Q498R, N501Y, and Y505H were incorporated into the original resolved crystal structure (PDB ID: 7A91) [21] using the Mutagenesis module of PyMOL software (http://www.pymol.org/pymol, accessed on 16 December 2021) to obtain the Omicron spike RBD model structure. The mutated structure was subjected to energy minimization using the conjugate gradient method in PyMOL software to remove any strains, and then used for molecular dynamics (MD) simulation and binding free energy studies.

### 3.2. Molecular Dynamics Simulation and Molecular Mechanics-Generalized Born Surface Area Free Energy Calculations

Molecular dynamics simulations of the protein complexes, (i) Omicron-RBD:hACE2, and (ii) wt-RBD:hACE2, were carried out using the AMBER 18.0 package [22,23] where f14SB force field [24] parameters were applied for proteins. The complexes were solvated with 37,000 water molecules (TIP3P force-field has been used to describe water) and neutralized with counterions in an orthorhombic simulation box [25]. The number of water molecules is decided by the cut-off distances along x, y, and z directions from the edge atoms of the protein-protein complex. In this case, a cut off distance of 10 Å was used for solvating the complex. The size of the whole system was approximately 140,000 atoms. The solvated systems were first energy-minimized. The energy minimization in Amber18 software uses the steepest descent and conjugate gradient algorithms, which use the derivatives of energies with respect to coordinates to find the local minimum in the potential energy surface. Subsequently, low-temperature simulations (at 30 K and 1 atm pressure) and MD simulations in ambient conditions (300 K and 1 atm) were carried out. The systems were allowed to equilibrate by running a short simulation of 10 ns. The time step for solving Newton’s equation of motion was set to 2 fs. The time scale for the production runs for each complex was set to 100 ns, and the trajectories were recorded every 50 ps time interval. Further, the trajectories were used for computing various properties such as RMSF, the radius of gyration (Rg), etc. The RMSF and Rg results computed for the spike protein and the hACE2 receptor are shown in Figures S1 and S4, respectively. The intermolecular non-covalent interactions such as hydrogen bonding, salt bridges, and pi-stacking were analyzed using VMD [26] and UCSF Chimera software [27]. The binding free energies were computed as an average over 2500 configurations corresponding to the last 10 ns of the production run using a molecular mechanics-generalized Born surface area approach (MM-GBSA) [28,29]. We also carried out the residue-wise decomposition analysis of binding free energies, and the results are presented in Figure 3.

## 4. Conclusions

The RBM sequence analysis and protein-protein interaction studies of the RBD:hACE2 complex, using molecular dynamics and binding free energy calculations, provided a detailed molecular level binding interaction pattern. The sequence and structural level investigation of the RBD revealed that the mutated residual composition in the Omicron variant exhibited an increase in the number of charged amino acids such as arginine, lysine, and aspartic acid that contribute to electrostatic interactions in proteins. Our investigation indicates that the mutated RBM interface bound tightly to ACE2 in the context of binding free energies. We have observed extra salt bridges between the RBM-R493 -- D30-ACE2, the RBM-R493 -- E35-ACE2, and the RBM-R493 -- D38-ACE2 in the Omicron variant. Interestingly, the lysine residue at position 417 in the RBD of the original SARS-CoV-2, which forms a salt-bridge with D30 of hACE2, did not show any interaction with this receptor after being mutated to glutamine in Omicron. Additionally, RBM’s mutated resides formed some additional hydrogen bonding and pi-stacking interactions, which could further enhance hACE2 binding. This information could be particularly informative for scientists of viral communities engaged in finding better therapeutics for SARS-CoV-2, especially for the new variants.

## Figures and Tables

**Table 1:** Binding free energies of the Omicron spike RBD and hACE2 complex.

System	ΔE_vdw_	ΔE_elec_	ΔE_psol_	ΔE_npsol_	ΔG, kcal/mol
Wild Type	−85.28	−856.42	922.17	−12.91	−32.43
B.1.1.529	−100.84	−1714.73	1787.97	−13.39	−41.00

The terms, ΔE_vdw,_ ΔE_elec,_ ΔE_psol_, and ΔE_npsol_ refer to van der Waals energy, electrostatic energy, polar solvation free energy, and non-polar solvation free energy, respectively. The term ΔG refers to the binding free energy for the spike protein with the hACE2 receptor.

**Table 2:** Hydrogen bonds and their occupancies and salt bridge ion-pairs found between spike RBM and hACE2 receptor during the entire MD simulation. The mutated residues in the Omicron variant are shown in bold.

No.	Wild Type-RBM: hACE2	H-Bond Occupancy (%)	Omicron-RBM: hACE2	H-Bond Occupancy (%)	Salt Bridges (Wild Type-RBM--hACE2)	Salt Bridges (Omicron-RBM--hACE2)
1	Tyr449-Asp38	70.50	Tyr449-Asp38	17.10	Lys417- -Asp30	**Arg493**- -Asp30
2	Thr500-Asp355	38.10	Thr500-Asp355	54.20		**Arg493**- -Glu35
3	Thr500-Tyr41	18.40	-	-		**Arg498**- -Asp38
4	Gln493-Glu35	38.20	**Arg493-**Asp30	39.20		
5	-	-	**Arg493-**Glu35	11.10		
6	Gln498-Asp38	26.90	-	-		
7	Gln498-Lys353	20.80	**Arg498-**Asp38	28.80		
8	Gly502-Lys353	48.60	Gly502-Lys353	48.60		
9	Tyr505-Glu37	22.70	**His505-**Ala386	16.50		
10	Gly496-Lys353	14.00	**Ser496-**Asp38	16.10		
11	Ser494-His34	14.90	Ser494**-**His34	12.20		
12	Asn487**-**Tyr83	28.30	Asn487-Tyr83	9.10		
13	Ala475-Gln24	11.10				
14	Lys417-Asp30	33.50	-	-		
15		-	Asn487-Gln24	13.90		
16	Gly446-Gln42	9.50	-	-		

## Data Availability

Data sharing not applicable.

## Supplementary Materials

The following are available online at https://www.mdpi.com/article/10.3390/ijms23063409/s1.
