# Supplementary material for: Improved Binding Affinity of Omicron’s Spike Protein for the Human Angiotensin-Converting Enzyme 2 Receptor Is the Key behind Its Increased Virulence
Source: Int J Mol Sci. 2022 Mar 21;23(6):3409. doi: 10.3390/ijms23063409 (PMC8955673; doi:10.3390/ijms23063409)
Supplement: Supplementary file 1 [file ijms-23-03409-s001.zip › ijms-1550155-supplementary.pdf]

Supporting information:

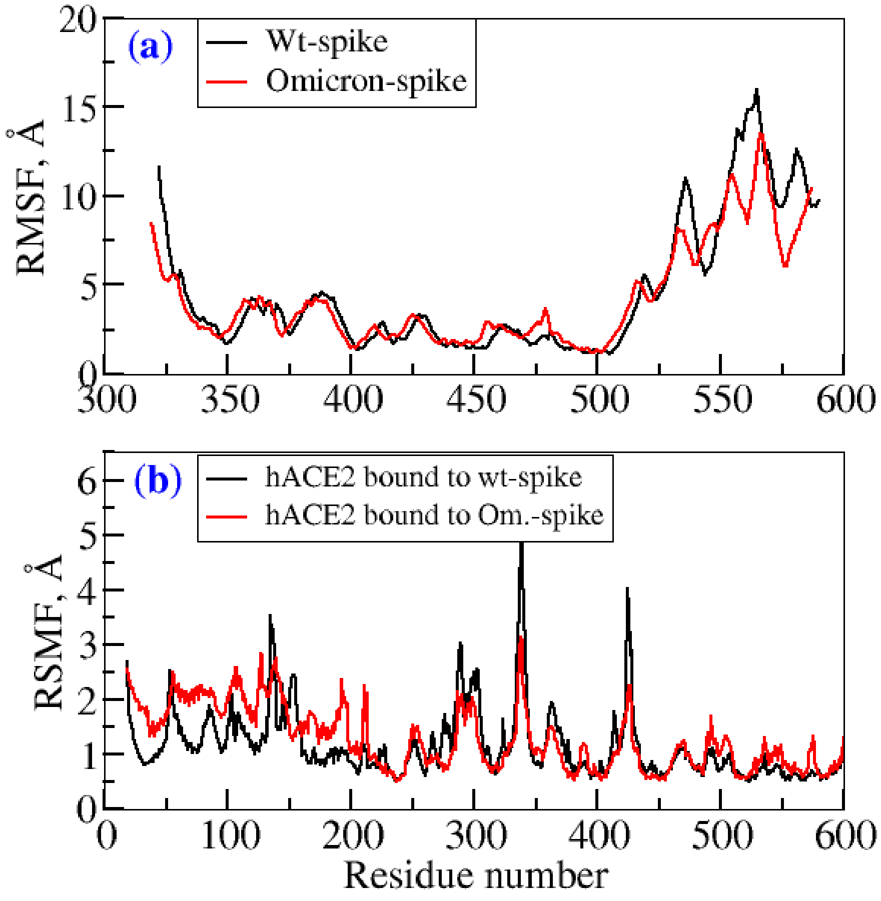

Figure S1. RMSFs computed for (**a**) spike protein and (**b**) hACE2 in the two complexes wt-spike: hACE2 and Omicron-spike: hACE-2.

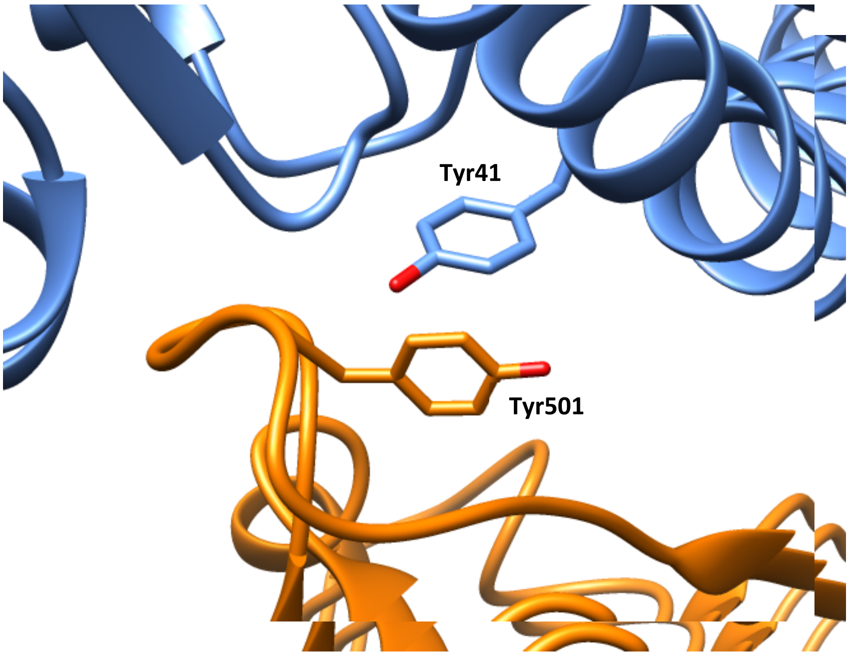

Figure S2. T-shape (edge-to-face) geometry of pi-pi stacking interaction between the two tyrosine residues in the spike:hACE2 complex, observed during MD simulation. The blue colour represents hACE2, and orange represents Omicron spike protein.

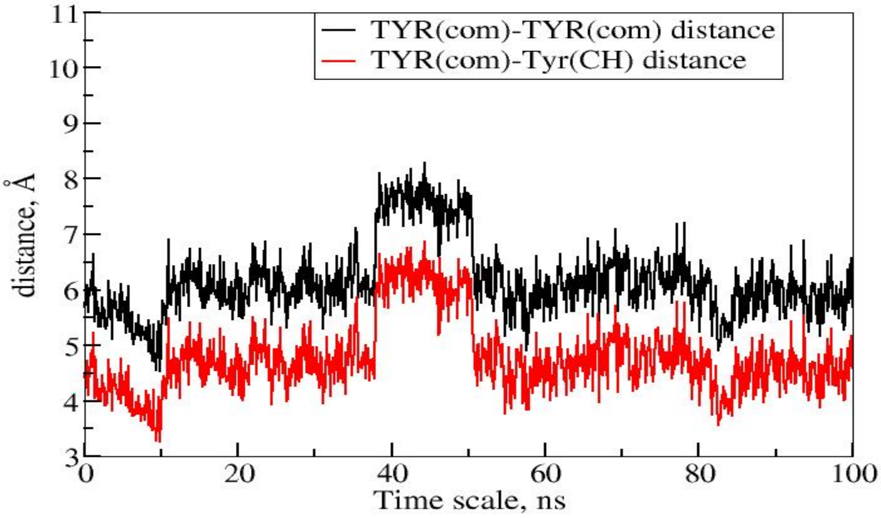

Figure S3. The time evolution of center of mass (COM) distance between Tyr 41 (hACE2) and Tyr501 (spike protein). The red colour represents the distance between the aromatic ring centroid of Tyr41 (hACE2) and the nearest H atom (HE) of Tyr501 (spike protein). The black colour represents the distance between the aromatic ring centroid of Tyr 41 (hACE2) and center of mass of edge-to-face carbon atoms of Tyr501 phenyl group (spike protein).

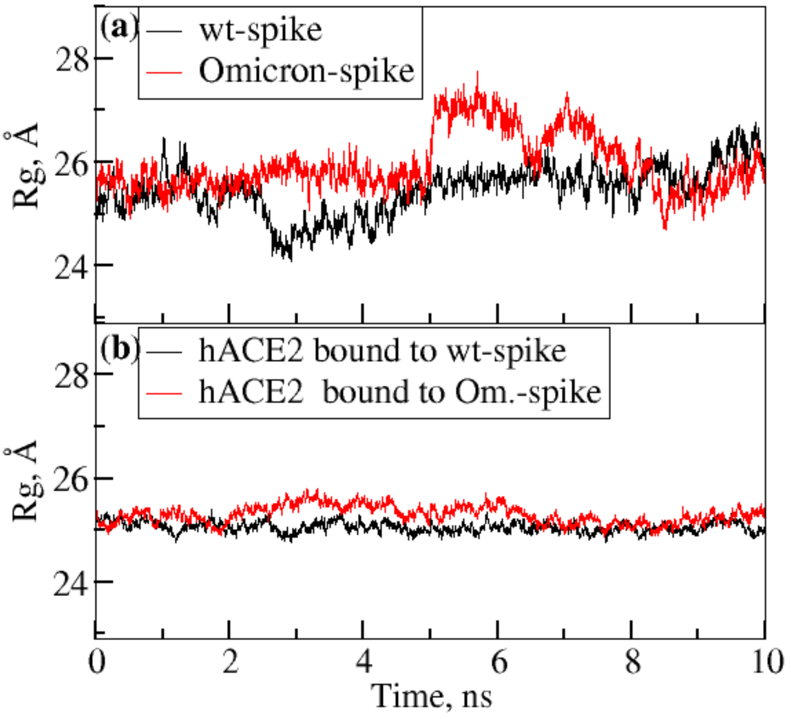

Figure S4. Radius of gyration computed for (**a**) the spike protein and (**b**) the hACE2 receptor.
